# Waitlist Mortality and Posttransplant Outcomes in African Americans with Autoimmune Liver Diseases

**DOI:** 10.1155/2021/6692049

**Published:** 2021-08-03

**Authors:** John Paul Nsubuga, Daniela Goyes, Hirsh D. Trivedi, Esli Medina-Morales, Vilas Patwardhan, Alan Bonder

**Affiliations:** ^1^Department of Medicine, Beth Israel Deaconess Medical Center, Boston, MA, USA; ^2^Division of Gastroenterology and Hepatology, Beth Israel Deaconess Medical Center, Boston, MA, USA

## Abstract

**Background:**

Liver transplantation is indicated in end-stage liver disease due to autoimmune diseases. The liver allocation system can be affected by disparities such as decreased liver transplant referrals for racial minorities, especially African Americans that negatively impact the pre- and posttransplant outcomes.

**Aim:**

To determine differences in waitlist survival and posttransplant graft survival rates between African American and Caucasian patients with autoimmune liver diseases. *Study*. The United Network for Organ Sharing database was used to identify all patients with autoimmune hepatitis, primary biliary cholangitis, and primary sclerosing cholangitis who underwent liver transplant from 1988 to 2019. We compared waitlist survival and posttransplant graft survival between Caucasians and African Americans using Kaplan–Meier curves and Cox regression models. We also evaluated the cumulative incidence of death or delisting for deterioration and posttransplant incidence of death and retransplantation using competing risk analysis.

**Results:**

African Americans were more likely to be removed from the waitlist for death or clinical deterioration (subdistribution hazard ratio (SHR) 1.26, 95% CI 1–1.58, *P*=0.046) using competing risk analysis. On multivariate Cox regression analysis, there was no difference in posttransplant graft survival among the two groups (hazard ratio (HR) 1.10, 95% CI 0.98–1.23, *P*=0.081).

**Conclusions:**

Despite the current efforts to reduce racial disparities, we found that African Americans are more likely to die on the waitlist for liver transplant and are less likely to be transplanted, with no differences in graft survival rates. The persistence of healthcare disparities continues to negatively impact African Americans.

## 1. Introduction

Autoimmune liver diseases (AILD) are immune-mediated diseases of the liver that are known to cause significant morbidity and mortality. They consist of three distinct entities, which include autoimmune hepatitis (AIH), primary biliary cholangitis (PBC), and primary sclerosing cholangitis (PSC). It has been proposed that these diseases are a result of interactions between genetic and environmental factors and nearly 10 percent of the cases are known to have overlapping syndromes which can make diagnosis challenging [1, 2]. Patients with AILD can progress to develop advanced hepatic fibrosis, which may in turn predispose them to the sequelae of decompensated liver cirrhosis including hepatocellular carcinoma (HCC), variceal hemorrhage, and ascites [3]. Although the use of immunosuppression in AIH and ursodeoxycholic acid and obeticholic acid in PBC has been shown to slow progression of these two diseases [4–7], no current treatments have been shown to slow progression of PSC [8]. Furthermore, a significant number of patients with AILD do progress despite treatment to end-stage liver disease requiring liver transplantation (LT). However, despite overall favorable outcomes of LT in AILD, healthcare disparities are present and negatively impact racial minority groups such as African Americans (AA). Such direct effect often impairs their ability to receive LT when indicated [9].

AA account for nearly 13 percent of the U.S. population and make up about 7.4% of the liver transplant waitlist [10]. Over time, AA continue to be overburdened by healthcare disparities when compared to other races. For instance, AA patients are less likely to be diagnosed with early-stage HCC and less likely to receive the early potentially curative treatments compared to Caucasian patients due to inadequate HCC surveillance [11]. Other studies also demonstrated that AA are less likely to undergo living donor liver transplantation due to fewer donation inquiries compared to other ethnic groups [12]. Furthermore, these differences varied depending on the etiology of the underlying liver disease [13]. However, research in evaluating potential disparities present in AA with AILD is lacking. We therefore aim to evaluate the differences in waitlist survival and posttransplant graft survival rates between AA and Caucasian patients using the United Nations Organ Sharing (UNOS) database.

## 2. Methods

### 2.1. Study Population

The UNOS Standard Transplant Analysis and Research (STAR) database was used to identify all patients with a primary listing diagnosis of AIH, PBC, and PSC who underwent LT in the United States from 1988 to 2019. We excluded children recipients (age <18 years), Hispanic/Latino, races other than Caucasian and AA, overlap syndromes (more than one underlying diagnosis for LT), those who had status 1 designation, and those listed for combined liver and kidney transplant. Furthermore, we excluded Hispanic/Latino patients given the known genetic heterogeneity associated with this population [14].

### 2.2. Definition of Outcomes

The primary outcomes were (1) waitlist survival using the composite outcome of death or removal for clinical deterioration and (2) posttransplant graft survival using the composite of posttransplant death or need for retransplantation. We also performed a subgroup analysis assessing posttransplant death alone or need for retransplantation alone.

### 2.3. Statistical Analysis

Recipient race was used to stratify clinical and demographic characteristics. Continuous variables were reported as median interquartile range (IQR) and were compared using Kruskal–Wallis test. Categorical variables were summarized using percentages and compared using Pearson's chi-squared test (*χ*^2^).

Kaplan–Meier curves and Cox regression models were used to compare patient survival on the waitlist and posttransplant graft survival among Caucasians and AA. Univariate analysis was performed for each variable to determine which covariates would be included in the adjusted model. Variables with a *P* < 0.10 in the univariate analysis and those of clinical significance were included in the model. For patient's survival on the waitlist, we adjusted for recipient age, sex, race, education, payment source, HCC diagnosis, blood type, MELD score at listing, and region in which the patient was listed. Competing risk analysis was used to evaluate the cumulative incidence of death or delisting for deterioration with liver transplant as a competing risk. For posttransplant survival, the final model included recipient characteristics such as age, gender, race, body mass index at transplant, and MELD score at transplant. We also adjusted for graft characteristics including cold ischemia time, donor age, and degree of ABO matching. Competing risk analysis was used to evaluate the cumulative incidence of death or retransplantation with retransplantation and death as competing risks, respectively. Trends over time were analyzed using the Cochran–Armitage test. All statistical analyses were conducted using Stata version 14.0 (College Station, TX StataCorp LP).

## 3. Results

### 3.1. Study Population Characteristics

A total of 24,493 patients with diagnosis of autoimmune liver disease (AIH, PBC, and PSC) who received a LT between 1988 and 2019 were identified.

Baseline characteristics for the respective groups are displayed in Table 1. Caucasians recipients accounted for 86.6% (*n* = 21,232), and AA for 13.3% (*n* = 3,261). A large proportion of AA recipients were female (64% vs. 58%, *P* ≤ 0.001) and were younger at the time of listing (43 (IQR, 31–54) vs. 53 (43–60), *P*=0.001). A higher percentage of AA had no college education (33% vs. 30%, *P* ≤ 0.001) and more often had public insurance (37% vs. 24%, *P* ≤ 0.001). They also had characteristics suggestive of more severe disease, including higher mean MELD scores at transplant (23 (IQR, 15–32) vs. 18 (IQR, 13–27), *P*=0.0001) and more comorbidities such as diabetes (17%), encephalopathy (33%), and ascites (45%) when compared to Caucasians.

PSC was the most common autoimmune liver disease among AA, followed by AIH and PBC (47%, 38%, and 14%, respectively). PSC was also the most common liver disease that afflicted Caucasians followed by PBC and AIH (45%, 32%, and 23%, respectively) (*P* < 0.001).

### 3.2. Waitlist Survival

**Figure 1:**
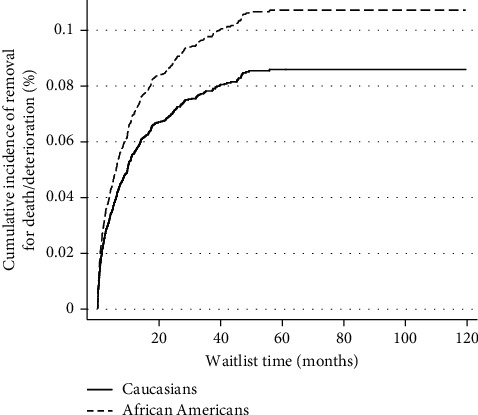
Competing risk analysis for cumulative incidence of death or waitlist removal for clinical deterioration with transplant as competing risk.

On multivariate Cox regression analysis, AA were more likely to be removed for death or clinical deterioration (hazard ratio (HR), 95% confidence interval (CI) 1.02–1.59, *P*=0.028) (Table 2). Likewise, on competing risk analysis with transplant as a competing risk, AA patients were more likely to be removed from the waitlist for death or clinical deterioration (subdistribution hazard ratio (SHR) 1.26, 95% CI 1–1.58, *P*=0.046) (Table 3, Figure 1).

### 3.3. Graft Survival Time

**Figure 2:**
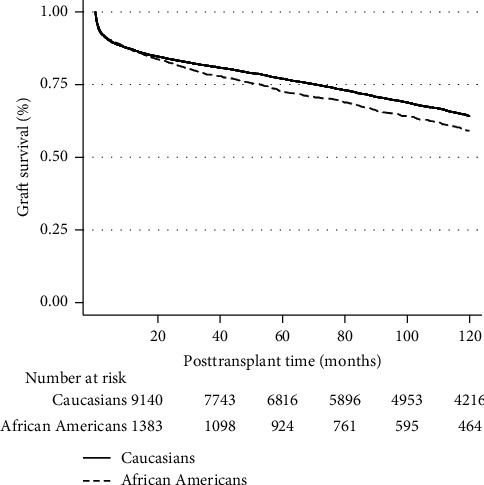
Unadjusted Kaplan–Meier estimates comparing graft survival (composite of posttransplant death and retransplant by race).

**Figure 3:**
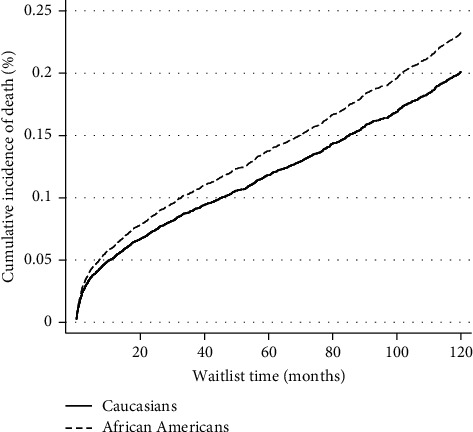
Competing risk analysis for cumulative incidence of death with retransplantation as competing risk.

**Figure 4:**
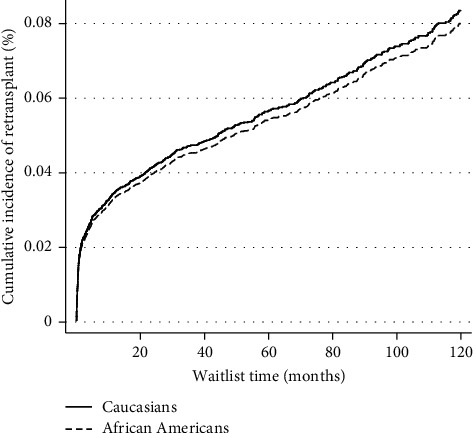
Competing risk analysis for cumulative incidence of retransplantation with death as competing risk.

On unadjusted analysis, AA patients had lower graft survival rates when compared to Caucasians status after LT (Figure 2). However, on multivariate Cox regression analysis, there was no difference in posttransplant graft survival among groups (HR 1.10, 95% CI 0.98–1.23, *P*=0.081) (Table 4). On multivariate competing risk analysis of the risk of death with retransplantation as a competing risk, AA patients were associated with increased risk of posttransplant death (HR 1.16, 95% CI 1.02–1.34, *P*=0.024) (Figure 3, Table 5). On competing risk analysis of the cumulative incidence of retransplantation with death as a competing risk, there was no difference among the two groups (SHR 0.97, 95% CI 0.81–1.17, *P*=0.824) (Figure 4, Table 5).

### 3.4. Trends in Transplant and Retransplant

We examined trends in LT and retransplant over time by ethnicity. The total transplants for AAs have increased over time from 9% in the period from 1988 to 1998 to 12% in 2008 to 2018 (*P* ≤ 0.001). Similarly, the percentage of AA patients undergoing retransplant has increased over time (*P*=0.001) (Table 6).

## 4. Discussion

Despite the increasing LT trends, better surgical procedures, and postoperative care, studies have demonstrated persistent disparities in LT outcomes by race [13, 15]. These disparities varied depending on the etiology of the underlying liver disease [13]. However, none of these studies have focused on AILD in AA. Our study shows that AA patients are an independent predictor of pre- and posttransplant survival outcomes in AILD.

Our multivariate analysis, which took into consideration noticeable differences in baseline characteristics, demonstrated that AA had an increased risk of delisting due to death or clinical deterioration. Similar findings have been reported previously in the literature. For instance, in a 2004 study by Reid et al., AA were more likely to die or become too ill for transplantation, and they were less likely to be transplanted within four years than Caucasians [16]. Furthermore, Echkoff et al. interestingly found that a higher proportion of AA compared to Caucasian patients were suitable and listed for transplantation (51% vs. 43%); however, a higher percentage of AA patients died waiting for a LT [17]. Our AA population had higher MELD scores at transplant that relates to overall worse disease severity and increased mortality. This is comparable to other studies that show that AA continue to carry a heavier burden of end-stage liver disease when compared to Caucasian patients [18]. This disproportion could be a reflection of other underlying medical conditions, late referral to transplant centers [17], poor socioeconomic status, and lack of insurance benefits resulting in poor compliance and inadequate management of the primary disease [19]. Even though studies during the post-MELD score era [20, 21] have demonstrated no significant disparities in transplant rates and waitlist outcomes between AA when compared to similar Caucasian candidates, these authors did not address disparities that are present in AA with AILD [22].

Despite worse pretransplant outcomes, our multivariate analysis shows that there is no difference in posttransplant graft survival rates among the two groups. Our findings are comparable to other reports indicating similar patient and graft survival rates between AA and Caucasian patients [17]. Perhaps, these results could be explained by the introduction of more potent immunosuppression therapies along with more frequent assessment of drug concentration and dose adjustments. For example, previous studies have shown an elimination of allograft survival differences among AA and Caucasians after the adoption of quadruple immunosuppression after renal transplant [23]. Furthermore, AILD have excellent outcomes for graft and patient survivals with reported 5-year and 10-year rates of approximately 90% to 75%, respectively [3]. Finally, research has shown Hispanic/Latino patients experience better posttransplant outcomes when compared to other ethnicities [24]. The removal of this patient population may have uncovered the lack of difference in graft survival between AA and Caucasian patients with AILD.

Nevertheless, in our competing analysis, AA patients were found to have increased risk of posttransplant death, independent of retransplantation rates. The cause of increased posttransplant deaths experienced by AA is not entirely understood; however, underlying comorbidities such as diabetes and its associated complications could play an important role [25, 26]. Preexisting diabetes has been shown to result in poor transplant outcomes. For example, in a large-scale study of diabetic patients undergoing a LT, patients with type 1 diabetes were found to have lower 5-year patient and graft survival rates [25]. As we mentioned earlier, socioeconomic barriers such as level of healthcare literacy, financial ability to pay for medication copays may also play a role as potential risk factors for noncompliance and increased risk of death in the posttransplant period [27].

The strength of this study is the utilization of a large database and its inclusion of more diverse subsets of patients which allows for better reflections of the disparities that may negatively impact AA patients during the pre- and posttransplant phases of LT. However, as in any study utilizing a large database, our study has several limitations. First, the respective nature of this study is limited by the available data present in the dataset, as such unmeasurable cofounding variables could be present despite our best efforts to account for all possible cofounding factors. Second, we did not have information on posttransplant variables such as immunosuppressive therapy and its adherence, which may also impact patient survival. Finally, we could not assess disease recurrence and cause of death after LT due to excess missing data.

## 5. Conclusion

In summary, our results show that healthcare disparities continue to negatively impact racial minorities, especially AA, who have worse waitlist outcomes and are less likely to be transplanted despite the increased knowledge of AILD. However, once transplanted, there is no difference in graft survival. We propose that the introduction of more potent immunosuppression therapies with close patient monitoring and frequent medication adjustments could explain lack of differences in graft survival rates. Further investigative studies are needed to assess whether pretransplant racial disparities are due to patient behavior, socioeconomic characteristics, or biological factors to ensure equal access and to detect inequalities in the allocation scarce liver organs.

## Figures and Tables

**Table 1:** Baseline characteristics.

Recipient characteristics			
	Caucasians, *n* = 21232	African Americans, *n* = 3261	*P* value
Gender, *n* (%)			
Male	8934 (42)	1163 (35.6)	<0.001
Female	12298 (57.9)	2098 (64.3)	
Age, median (IQR)	53 (43–60)	43 (31–54)	0.001
BMI, median (IQR)	25.5 (22.6–29.2)	25.7 (22.6–30.5)	0.002
BMI ≥30, *n* (%)	4930 (23.2)	925 (28.3)	<0.001
Blood type, *n* (%)			
A	8702 (40.9)	845 (25.9)	<0.001
B	2241 (10.5)	663 (20.3)	
AB	722 (3.4)	135 (4.1)	
O	9567 (45)	1618 (49.6)	
Public insurance, *n* (%)	5038 (23.7)	1207 (37)	<0.001
No college education, *n* (%)	6337 (29.8)	1083 (33.2)	<0.001
Comorbidities, *n* (%)			
Diabetes	2552 (12)	540 (16.5)	<0.001
Encephalopathy	6535 (30.7)	1079 (33)	<0.001
Ascites	8803 (41.4)	1464 (44.8)	<0.001
Muscle wasting	1682 (7.9)	171 (5.2)	<0.001
SBP	914 (4.3)	140 (4.2)	0.006
HCC diagnosis ever, *n* (%)	185 (0.87)	23 (0.71)	<0.001
Functional status at listing, *n* (%)			
No assistance	10861 (51.1)	1421 (43.5)	<0.001
Some assistance	6206 (29.2)	1023 (31.3)	
Total assistance	1782 (8.3)	459 (14)	
Missing	2383 (11.2)	358 (10.9)	
Level of care, *n* (%)			
Outpatient	8691 (40.9)	1236 (37.9)	<0.001
Inpatient non-ICU	2085 (9.8)	413 (12.6)	
Inpatient ICU	1312 (6.1)	257 (7.8)	
Missing	9144 (43)	1355 (41.5)	
Primary diagnosis, *n* (%)			
AIH	4935 (23.2)	1254 (38.4)	<0.001
PBC	6796 (32)	466 (14.2)	
PSC	9501 (44.7)	1541 (47.2)	
Waitlist days, median (IQR)	242 (65–734)	178 (31–626)	0.0001
UNOS region			
1	1081 (5)	63 (1.9)	<0.001
2	2608 (12.2)	514 (15.7)	
3	2211 (10.4)	646 (19.8)	
4	1867 (8.7)	335 (10.2)	
5	2781 (13.1)	199 (6.1)	
6	784 (3.6)	23 (0.71)	
7	2452 (11.5)	233 (7.1)	
8	2087 (9.8)	139 (4.2)	
9	1268 (5.9)	312 (9.5)	
10	2250 (10.6)	306 (9.3)	
11	1843 (8.6)	491 (15)	
MELD score at transplant, median (IQR)	18 (13–27)	23 (15–32)	0.0001
Donor characteristics			
Age, median (IQR)	39 (24–52)	37 (23–51)	0.0174
Type, *n* (%)			
Deceased	10903 (51.3)	1834 (56.2)	<0.001
Living	1232 (5.8)	79 (2.4)	
Missing	9097 (42.8)	1348 (41.3)	
Cold ischemia, median (IQR)	6.5 (4.7–8.8)	6.3 (4.8–8.5)	0.5940
ABO, *n* (%)			
Matched	11220 (52.8)	1743 (53.4)	0.043
Compatible	823 (3.8)	149 (4.5)	
Incompatible	90 (0.42)	21 (0.65)	
Missing	9099 (42.8)	1348 (41.3)	

BMI, body mass index. HCC, hepatocellular carcinoma. SBP, spontaneous bacterial peritonitis. ICU, intensive care unit. AIH, autoimmune hepatitis. PBC, primary biliary cholangitis. PSC, primary sclerosing cholangitis. MELD, model for end-stage liver disease. IQR, interquartile range.

**Table 2:** Multivariate Cox proportional hazards model for waitlist survival.

	HR	95% CI	*P* value
Age	1.04	1.04–1.05	<0.001
Gender (female)	0.75	0.63–0.89	0.001
Presence of HCC	1.38	0.92–2.06	0.114
Initial MELD	1.17	1.16–1.18	<0.001
No college education	1.04	0.88–1.24	0.577
Payment source			
Private	Ref		
Public	1.54	1.30–1.83	<0.001
Other	1.36	0.50–3.68	0.543
ABO group			
A	Ref		
B	0.80	0.59–1.06	0.131
AB	1.47	0.92–2.37	0.103
O	0.94	0.79–1.12	0.528
UNOS region			
1	Ref		
2	0.93	0.63–1.39	0.753
3	0.63	0.41–0.96	0.032
4	1	0.66–1.50	0.993
5	0.62	0.41–0.94	0.025
6	0.69	0.41–1.17	0.176
7	0.70	0.47–1.05	0.092
8	0.95	0.62–1.44	0.824
9	0.71	0.45–1.11	0.143
10	1.13	0.76–1.68	0.535
11	0.81	0.53–1.22	0.320
African Americans	1.28	1.02–1.59	0.028

MELD, model for end-stage liver disease. HCC, hepatocellular carcinoma. HR, hazard ratio. CI, confidence interval. REF, reference.

**Table 3:** Multivariate competing risk regression analysis for death or waitlist removal for clinical deterioration with transplant as a competing risk.

Variables	SHR	95% CI	*P* value
Age	1.03	1.02–1.04	<0.001
Gender (female)	0.71	0.60–0.85	<0.001
Presence of HCC	1.10	0.73–1.65	0.632
Initial MELD	1.05	1.04–1.06	<0.001
No college education	1.04	0.87–1.24	0.661
Payment source			
Private	REF		
Public	1.56	1.30–1.86	<0.001
Other	1.14	0.41–3.16	0.801
UNOS region			
1	REF		
2	0.76	0.51–1.14	0.190
3	0.38	0.25–0.58	<0.001
4	0.79	0.51–1.20	0.275
5	0.62	0.41–0.94	0.025
6	0.83	0.49–1.42	0.514
7	0.67	0.44–1.01	0.059
8	0.80	0.52–1.22	0.304
9	0.81	0.52–1.26	0.358
10	0.70	0.47–1.05	0.088
11	0.59	0.39–0.91	0.017
Blood type			
A	REF		
B	0.66	0.49–0.89	0.007
AB	0.71	0.43–1.17	0.189
O	0.90	0.76–1.08	0.290
African Americans	1.26	1–1.58	0.046

MELD, model for end-stage liver disease. HCC, hepatocellular carcinoma. SHR, subdistribution hazard ratio. CI, confidence interval. REF, reference.

**Table 4:** Multivariate Cox proportional hazards model for graft survival.

	HR	95% CI	*P* value
Age	1	0.99–1	0.300
Gender	1.16	1.09–1.27	0.001
BMI at transplant	0.99	0.99–1	0.907
MELD at transplant	1	1–1.01	0.004
Cold ischemia (hours)	1.02	1.01–1.03	<0.001
Donor age	1	1–1.01	<0.001
ABO match			
Identical	Ref		
Compatible	1.05	0.90–1.22	0.493
Incompatible	0.89	0.54–1.46	0.658
Diagnostic			
AIH	Ref		
PBC	0.82	0.73–0.93	0.001
PSC	0.82	0.74–0.92	0.001
African Americans	1.10	0.98–1.23	0.081

MELD, model for end-stage liver disease. HCC, hepatocellular carcinoma. SHR, subdistribution hazard ratio. CI, confidence interval. REF, reference. AIH, autoimmune hepatitis. PBC, primary biliary cholangitis. PSC, primary sclerosing cholangitis.

**Table 5:** Multivariate competing risk analysis for graft survival.

	Death with retransplantation as competing risk			Retransplantation with competing risk of death		
	SHR	95% CI	*P* value	SHR	95% CI	*P* value
Age	1.02	1.02–1.03	<0.001	0.95	0.95–0.96	<0.001
Gender	1.18	1.06–1.31	0.002	1.06	0.90–1.24	0.475
BMI at transplant	1	1.02–1.34	0.024	1	0.98–1	0.824
MELD at transplant	1	1–1	0.001	0.99	0.98–1	0.301
Cold ischemia (hours)	1.02	1–1	<0.001	1.02	1–1.04	0.002
Donor age	1	1–1	0.002	1.01	1.01–1.02	<0.001
ABO match						
Identical	Ref					
Compatible	0.99	0.82–1.2	0.968	1.16	0.89–1.51	0.256
Incompatible	1.08	0.62–1.88	0.782	0.56	0.17–1.81	0.337
Diagnostic						
AIH	Ref					
PBC	0.77	0.68–0.88	<0.001	1.04	0.81–1.34	0.723
PSC	0.69	0.61–0.79	<0.001	1.32	1.09–1.61	0.005
African Americans	1.16	1.02–1.34	0.024	0.97	0.81–1.17	0.824

BMI, body mass index. AIH, autoimmune hepatitis. PBC, primary biliary cholangitis. PSC, primary sclerosing cholangitis. MELD, model for end-stage liver disease. SHR, subdistribution hazard ratio. CI, confidence interval. REF, reference.

**Table 6:** Trends in proportion of total liver transplant by race over time.

	1988–1998	1999–2008	2009–2018	Total (*n*)
Caucasians (%)	91	88	83	11,522
African Americans (%)	9	12	17	1,804
Total transplants (n)	2,914	5,293	5,119	13,326
*Z* = 10.26, *P* ≤ 0.001				

*Trends in proportion of total retransplant by race over time*				
	1988–1998	1999–2008	2009–2018	Total (n)

Caucasians (%)	93	84	82	1,152
African Americans (%)	7	16	18	214
Total retransplants (*n*)	229	574	563	1,366
*Z* = 3.47, *P*=0.001				

## Data Availability

The data used to support the findings of this study may be released upon application to the Organ Procurement and Transplantation Network, who can be contacted at https://optn.transplant.hrsa.gov/data/request-data/.

## Abbreviations

AIH: Autoimmune hepatitis
AILD: Autoimmune liver diseases
AA: African Americans
BMI: Body mass index
CI: Confidence interval
HCC: Hepatocellular carcinoma
HR: Hazard ratio
ICU: Intensive care unit
IQR: Interquartile range
LT: Liver transplant
MELD: Model for end-stage liver disease
PBC: Primary biliary cirrhosis
PSC: Primary sclerosing cholangitis
REF: Reference
SBP: Spontaneous bacterial peritonitis
SHR: Subdistribution hazard ratio
UNOS: United Network of Organ Sharing.
